# OptBand: optimization-based confidence bands for functions to characterize time-to-event distributions

**DOI:** 10.1007/s10985-021-09522-8

**Published:** 2021-04-13

**Authors:** T. Chen, S. Tracy, H. Uno

**Affiliations:** 1 Department of Population Medicine, Harvard Medical School and Harvard Pilgrim Health Care, Boston, USA; 2 Department of Biostatistics, Harvard T.H. Chan School of Public Health, Boston, USA; 3 Department of Data Science, Dana-Faber Cancer Institute, Boston, USA

**Keywords:** Survival, Confidence band, Highest density region, Optimization

## Abstract

Classical simultaneous confidence bands for survival functions (i.e., Hall–Wellner, equal precision, and empirical likelihood bands) are derived from transformations of the asymptotic Brownian nature of the Nelson–Aalen or Kaplan–Meier estimators. Due to the properties of Brownian motion, a theoretical derivation of the highest confidence density region cannot be obtained in closed form. Instead, we provide confidence bands derived from a related optimization problem with local time processes. These bands can be applied to the one-sample problem regarding both cumulative hazard and survival functions. In addition, we present a solution to the two-sample problem for testing differences in cumulative hazard functions. The finite sample performance of the proposed method is assessed by Monte Carlo simulation studies. The proposed bands are applied to clinical trial data to assess survival times for primary biliary cirrhosis patients treated with D-penicillamine.

## Introduction

For time-to-event outcomes in clinical studies, inference for the cumulative distribution and survival functions are of interest. The Kaplan–Meier (KM) estimator and its corresponding asymptotic variance are often used to construct pointwise confidence intervals. When interest lies in inference of the survival curve over an entire time interval, “simultaneous” confidence bands (CB) need to be constructed instead of a set of pointwise confidence intervals.

For continuous function $\varphi (t)$ which characterizes the event time distribution, we say two stochastic processes $\mathfrak {L}(t)$ and $\mathfrak {U}(t)$ are $(1-\alpha )$ coverage level simultaneous CBs for $\varphi (t)$ on the interval $[t_L, t_U]$ if

$$\begin{aligned} \mathbb {P}\Big (\mathfrak {L}(t) \le \varphi (t) \le \mathfrak {U}(t) \quad \forall t \in [t_L, t_U]\Big ) \ge 1-\alpha . \end{aligned}$$

Common choices for $\alpha$ are 0.01, 0.05, 0.10. We distinguish this definition from the standard pointwise CBs, which are constructed such that at each time point *t*, $\mathbb {P}(\mathfrak {L}(t) \le \varphi (t) \le \mathfrak {U}(t)) = 1-\alpha$. Clearly, pointwise CBs are narrower than simultaneous CBs, but the former do not attain the nominal coverage level $(1-\alpha )$ on the specified interval $[t_L, t_U]$. Two classical simultaneous CBs based on the asymptotics of the KM estimator were originally developed by Hall and Wellner (1984) and Nair (1984); they are now popularly referred to as the Hall–Wellner (HW) and equal precision (EP) bands, respectively. Both of these aforementioned papers derived a pivotal quantity for the estimated survival function and showed that this quantity weakly converges to a Brownian motion or Brownian bridge, which were then inverted to obtain simultaneous CBs for the survival function. Hollander et al. (1997) derived simultaneous CBs for survival and cumulative hazard functions based on the empirical likelihood (EL) approach for confidence intervals introduced by Thomas and Grunkemeier (1975). This technique has also been applied to quantile functions by Li et al. (1996), but was not applied to the two-sample problem. Furthermore, McKeague and Zhao (2002) proposed CBs for ratios of survival functions using EL techniques. They also extended their approaches to construct CBs for differences and ratios of linear functionals of the cumulative hazard functions (McKeague and Zhao 2005). The extended approach relies on simulation to estimate critical values. Moreover, EL CBs for survival functions are not readily accessible as they have not been implemented in standard computing software. Parzen et al. (1994) introduced a perturbation resampling method as a generalized approach in calculating critical values for transformed distributions and notably applied this to the two-sample problem (Parzen et al. 1997). Tian et al. (2011) derived bands which target the highest confidence density region (HCDR), but this approach requires the standard specifications and tuning procedures that accompany a Markov chain Monte Carlo process and can be computationally burdensome. Finally, Cui and Hannig (2019a) introduced a nonparametric fiducial approach to confidence bands, which has been shown to be robust and efficient in small samples; several auxiliary works (Cui and Hannig 2019b; Martin 2019) explored the implications of this work and how fiducial inference fits in the context of modern statistics.

In this paper, we propose a novel procedure to construct analytical simultaneous CBs $\{\mathfrak {L}(t), \mathfrak {U}(t)\}$ for $\varphi (t)$ which approximately target the HCDR. That is, such bands aim to minimize

1 $$\begin{aligned} \begin{aligned}&{\mathbb {E}\left( \int _{t_L}^{t_U}\left\{ \mathfrak {U}(t) - \mathfrak {L}(t)\right\} dt\right) } \quad \\&\text {s.t.} \quad \mathbb {P}\Big (\mathfrak {L}(t) \le \varphi (t) \le \mathfrak {U}(t) \quad \forall t \in [t_L, t_U]\Big ) = 1-\alpha . \end{aligned} \end{aligned}$$

To accomplish the objective in Eq. (1), we utilize an analytical result from Kendall et al. (2007), which provides an approximate solution to a related optimization problem

2 $$\begin{aligned} \begin{aligned}&\min _{u}\left( \int _{t_L}^{t_U}u(t) dt\right) \\&\text {s.t.} \quad \mathbb {P}\Big (-u(t) \le W(\sigma ^2(t)) \le u(t) \quad \forall t \in [t_L, t_U]\Big ) = 1-\alpha . \end{aligned} \end{aligned}$$

The approximate solution to Eq. (2) is $u_\kappa ^*(t) = \psi (\kappa \sigma ^2(t))\sigma (t)$, where $W(\sigma ^2(t))$ is a mean-zero Brownian motion with strictly-increasing variance function $\sigma ^2(t)$, $\kappa$ is a critical value related to the desired coverage level $1-\alpha$, $\psi (x) = (-\mathcal {W}_{-1}(-x^2))^{1/2}\mathbb {I}(x \le e^{-1/2})$, $\mathcal {W}_{-1}$ is the Lambert *W* function on the $-1$ branch, and $\mathbb {I}$ is the indicator function (see Appendix A for details of the derivation). The solution $u_\kappa ^*(t)$ is approximate in the sense that it replaces the probability constraint in Eq. (2) with its local time portion from a Doob-Meyer decomposition. Nevertheless, Kendall et al. (2007) have demonstrated its success in practical contexts, and henceforth we coin our simultaneous CBs derived from their results as simply “OptBand”. To wit, our strategy is to form pivotal quantities that are asymptotically Brownian, apply the result by Kendall et al. (2007), and transform these pitoval quantities into CBs for $\varphi (t)$, which could be either the cumulative hazard function or survival function.

In practice, there are two challenges in adopting the work of Kendall et al. (2007). The first is the burden in calculating the critical value parameter $\kappa$. As shown in Appendix A, $\kappa$ depends on $t_L, t_U, \sigma ^2(t)$, and $\alpha$, which are typically study-specific and depend on the clinical research question of interest. Secondly, how to apply this theory to construct OptBand for survival functions is not entirely clear. Non-linear pivotal quantity transformations do not necessarily preserve the HCDR property from the optimization problem in Eq. (2) to the optimization problem in Eq. (1). The pivotal quantity transformation for the cumulative hazard function happens to be exactly linear and therefore it is straightforward to derive OptBand for this case. The survival function, however, requires more sophisticated means. In this paper, we tackle these challenges. In Sect. 2, we derive a highly accurate functional approximation in calculating $\kappa$ so that users may automatically determine $\kappa$ for their specific problems and not have to be constrained to specific parameters found in standard critical value tables. We construct OptBand for the one-sample cumulative hazard function and two-sample difference in cumulative hazard functions in Sect. 3. OptBand for the one-sample survival function is derived in Sect. 4. We evaluate OptBand’s performance against performances of fiducial, empirical likelihood, equal precision, and Hall-Wellner bands in Sect. 5, and illustrate with an application to clinical trial data concerning primary biliary cirrhosis in Sect. 6. The paper concludes with a discussion in Sect. 7.

## Functional approximation of the critical value parameter

Akin to the critical values required for the HW or EP bands, OptBand’s critical value, $\kappa$, is required to attain a desired $(1-\alpha )$ coverage level. Such value is computed to satisfy

$$\begin{aligned} \mathbb {P}\Big (|W(\sigma ^2(t))| \le \psi (\kappa \sigma ^2(t)/\sigma ^2(t_U))\sqrt{\sigma ^2(t)} \quad \forall t \in [t_L, t_U]\Big ) = 1-\alpha . \end{aligned}$$

Kendall et al. (2007) performed a Monte Carlo simulation for the case $t_L = 0$, $t_U = 1$, ${\alpha = 0.05}$, $\sigma ^2(t) = t$ (the standard Brownian motion on the unit interval) in approximating $\kappa \approx 0.105$. We provide an approximation procedure that generalizes their simulation.

Without loss of generality, we may consider minimizing the interval around $W(\sigma ^2(t)/\sigma ^2(t_U))$ for $t \in [t_L, t_U]$, since by properties of Brownian motion, $\sigma ^{-1}(t_U)W(\sigma ^2(t))$ and $W(\sigma ^2(t)/\sigma ^2(t_U))$ are equal in distribution. Hence, we only need to compute $\kappa$ such that

3 $$\begin{aligned} \mathbb {P}(|W(s)| \le \psi (\kappa s)s^{1/2} \quad \forall s \in [L, 1]) = 1-\alpha . \end{aligned}$$

Therefore, $\kappa$ is now only a function of $\alpha$ and $L \overset{\text {def}}{=} \sigma ^2(t_L)/\sigma ^2(t_U) \in [0, 1]$. Through Monte Carlo simulations calculations using Eq. (3), we form an array of $(1-\alpha )$ coverage values corresponding to $(\kappa , L)$. While this array is sufficient as a lookup table for the critical values, the functional relationship is accurately approximated with $1-\alpha \approx 1 + (a+bL)\kappa + a\kappa ^2$, or

4 $$\begin{aligned} \begin{aligned} \kappa \approx -\frac{ (a+bL)+\{(a + bL)^2 - 4a[1-(1-\alpha )]\}^{1/2} }{2a}. \end{aligned} \end{aligned}$$

where $a = -0.4272, b = 0.2848$ (see Appendix A). As a measure of goodness of fit, the adjusted $R^2$ for Eq. (4) based on our Monte Carlo data is 0.9987. Furthermore, the resulting $(1-\alpha )$ from the simulation ranges from 0.871 to 0.999, encompassing accurate interpolation for clinically relevant coverage levels of 0.90, 0.95, and 0.99. Applying Eq. (4) to the standard Brownian motion on [0, 1], we calculate $\kappa = 0.106$, which is very close to the value specified in Kendall et al. (2007).

## OptBand for the cumulative hazard function

### One-sample problem

Let *T* following distribution *F* and *C* following distribution *G* denote failure times and censoring times, respectively. Let $\{T_i, C_i\}_{i=1}^{n}$ be i.i.d. copies of $\{T,C\}$. Throughout the paper, we assume *T* and *C* are independent of each other. Our observed data are $(\widetilde{T}_i, \varDelta _i)_{i=1}^{n}$, where $\widetilde{T}_i = \min (T_i, C_i)$ and $\varDelta _i = \mathbb {I}\{T_i \le C_i\}$. Let $\widehat{H}(t)$ be the Nelson-Aalen estimator of the true cumulative hazard function *H*(*t*). Restricting our interval of interest to $[t_L, t_U]$, standard asymptotic results dictate that $n^{1/2}\{\widehat{H}(t) - H(t)\}/\sigma (t_U)$ weakly converges to $W\left( \sigma ^2(t)/\sigma ^2(t_U)\right)$ (Fleming and Harrington 1991), where

$$\begin{aligned} {\sigma ^2(t) = \int _{0}^{t} \frac{dH(s)}{(1-F(s))(1-G(s))}}. \end{aligned}$$

OptBand’s inference is based on the pivotal quantity

$$\begin{aligned} \left| n^{1/2}\frac{\widehat{H}(t) - H(t)}{\sigma (t_U)}\right| \le \psi \left( \kappa \frac{\sigma ^2(t)}{\sigma ^2(t_U)}\right) \frac{\sigma (t)}{\sigma (t_U)}. \end{aligned}$$

Since we do not observe the true variance $\sigma ^2(t)$, we replace it with an estimator. Throughout, we will use Greenwood’s formula

$$\begin{aligned} \widehat{\sigma }^2(t) = n \sum _{j: t_j \le t}\frac{d_j}{n_j(n_j - d_j)}, \end{aligned}$$

where $d_i = \sum _{j: T_j = \widetilde{T}_i}T_j\varDelta _j$ is the number of failures at $\widetilde{T}_i$, and $n_i = |\{j:T_j \ge \widetilde{T}_i\}|$ is the number of individuals at risk at time $\widetilde{T}_i$. Pivoting, the asymptotic $(1-\alpha )$ level OptBand for *H*(*t*) is $\lbrace \mathfrak {L}(t), \mathfrak {U}(t)\rbrace = \widehat{H}(t) \pm c_{CH}(t)$, where

$$\begin{aligned} c_{CH}(t) = \psi \left( \kappa \frac{\widehat{\sigma }^2(t)}{\widehat{\sigma }^2(t_U)}\right) \frac{\widehat{\sigma }(t)}{n^{1/2}} \end{aligned}$$

and $\kappa$ is computed using Eq. (4).

### Two-sample problem

The two-sample problem considers CBs for $H_1(t) - H_2(t)$, where $H_1(t)$ and $H_2(t)$ are the cumulative hazard functions which give rise to data $(\widetilde{T}_{1i}, \varDelta _{1i})_{i=1}^{n_1}$ and $(\widetilde{T}_{2i}, \varDelta _{2i})_{i=1}^{n_2}$, respectively. The quantity

$$\begin{aligned}{}[\widehat{H}_1(t) - \widehat{H}_2(t)]- [H_1(t)-H_2(t)]&= [\widehat{H}_1(t) - H_1(t)] - [\widehat{H}_2(t) - H_2(t)] \end{aligned}$$

weakly converges to $W\left( n_1^{-1}\sigma ^2_1(t) +n_2^{-1}\sigma ^2_2(t)\right)$. Applying the same strategy as before, the pivotal quantity must satisfy, with probability $(1-\alpha )$, as follows:

$$\begin{aligned} \left| (n_1+n_2)^{-1/2}\frac{[\widehat{H}_1(t) - H_1(t)] - [\widehat{H}_2(t) - H_2(t)]}{\sigma _p(t_U)}\right| \le \psi \left( \kappa \frac{\sigma ^2_p(t)}{\sigma ^2_p(t_U)}\right) \frac{\sigma _p(t)}{\sigma _p(t_U)}, \end{aligned}$$

where $\sigma ^2_p(t) = (n_1 + n_2)\left( \frac{\sigma _1^2}{n_1} + \frac{\sigma _2^2}{n_2}\right)$. Replacing $\sigma ^2_p(t)$ with estimator $\widehat{\sigma }^2_p(t)$ and pivoting, we derive the $(1-\alpha )$ level OptBand for $H_1(t)-H_2(t)$ as $\lbrace \mathfrak {L}(t), \mathfrak {U}(t)\rbrace = [\widehat{H}_1(t) - \widehat{H}_2(t)] \pm c_{2CH}(t)$, where

$$\begin{aligned} c_{2CH}(t) = \psi \left( \kappa \frac{\widehat{\sigma }^2_p(t)}{\widehat{\sigma }^2_p(t_U)}\right) \frac{\widehat{\sigma }_p(t)}{(n_1 + n_2)^{1/2}}. \end{aligned}$$

As before, $\kappa$ is computed using Eq. (4).

## OptBand for the survival function

### One-sample problem

Let $\widehat{S}(t)$ be either the Fleming-Harrington (Fleming and Harrington 1991) or Kaplan–Meier estimator for the true survival function *S*(*t*). Standard asymptotic results show that both estimators satisfy that

5 $$\begin{aligned} n^{1/2}\frac{\widehat{S}(t) - S(t)}{S(t)} \quad \text {weakly converges to} \quad W\left( \sigma ^2(t)\right) . \end{aligned}$$

As noted in the Introduction, we cannot apply the same strategy with this pivotal quantity as with cumulative hazard functions as it would focus on minimizing the area between the bands of $\{\widehat{S}(t) - S(t)\}/S(t)$, not $\widehat{S}(t) - S(t)$. We overcome this problem by weighting the Eq. (5) by *S*(*t*) and re-optimizing; that is, we target $\widetilde{u}(t) = S(t)u(t)$ so that $n^{1/2}|\widehat{S}(t) - S(t)| \le \widetilde{u}(t)$ with the same optimization strategy from Kendall et al. (2007). As shown in Appendix B, the $(1-\alpha )$ level OptBand for *S*(*t*) is $\lbrace \mathfrak {L}(t), \mathfrak {U}(t)\rbrace = \widehat{S}(t)\left( 1 \pm c_S(t)\right)$, where

$$\begin{aligned} c_S(t) = \psi \left( \kappa \widehat{S}(t)\frac{\widehat{\sigma }^2(t)}{\widehat{\sigma }^2(t_U)}\right) \frac{\widehat{\sigma }(t)}{n^{1/2}} \end{aligned}$$

and $\kappa$ is calculated according to

$$\begin{aligned} \begin{aligned} \kappa = -\frac{\tilde{b} + \{\tilde{b}^2 - 4\tilde{a}\tilde{c}\}^{1/2}}{2\tilde{a}} \end{aligned} \end{aligned}$$

to obtain a coverage level of $1-\alpha$, where

$$\begin{aligned} \tilde{a}&= a\overline{S}_{K-1}^2, \\ \tilde{b}&= \frac{b}{\widehat{\sigma }^2(t_U)}\left\{ \sum _{i=1}^{K-2}\overline{S}_i(\widehat{\sigma }^2(\xi _i) - \widehat{\sigma }^2(\xi _{i+1}))\right\} + \left( a + b \frac{\widehat{\sigma }^2(\xi _{K-1})}{\widehat{\sigma }^2(t_U)}\right) \overline{S}_{K-1}, \\ \tilde{c}&= \alpha , \end{aligned}$$

and $\overline{S}_i = (\widehat{S}(\xi _i) + \widehat{S}(\xi _{i+1}))/2$, where $\xi _1 \le \xi _2 \le \cdots \le \xi _K$ are the failure time points we observe in the sample and *K* is the total number of observed points (see Appendix B for derivation).

## Simulation

We compare our proposed OptBand against FD-I (Cui and Hannig 2019a), EL (Hollander et al. 1997), EP (Nair 1984), and HW (Hall and Wellner 1984) bands. Hollander et al. (1997) proposed several types of EL bands; we compare against a bias-corrected EP-type EL band, which was shown to have the most consistently good performance. We generate $$T_i $$ from a unit exponential distribution and $$C_i$$ from an exponential distribution with rate parameters 0, 0.25, 1 and 9 to demonstrate 0%, 20%, 50% and 90% censored observations, respectively. These were done at sample sizes $$n = 100, 500, 1000$$ across $$R = 2,000$$ replicate simulations. We restrict to time intervals $$\mathcal {T}_{a,b} = \{t: a \le d(t) \le b\}$$, where $$d(t) = \widehat{\sigma }^2(t)/[1 + \widehat{\sigma }^2(t)]$$ and (*a*, *b*) = (0.05, 0.95), (0.05, 0.8), (0.2, 0.8), (0.2, 0.95). This form of time restriction, as opposed to trimming off the first *a* and last $$1-b$$ quantiles of times, was the formulation used by Nair (1984) and Hollander et al. (1997) when evaluating the validity of the coverage of their bands. The form of *d*(*t*) is derived from a transformation used in Nair (1984) to yield EP bands. Critical values for EP bands were only established for $$0.02 \le a < b \le 0.98$$ (Klein and Moeschberger 2006), and both Nair (1984) and Hollander et al. (1997) recommended their methods to be used for $$0.05 \le a < b \le 0.95$$. Furthermore, we include time interval restrictions as part of the simulation scenarios to evaluate the regions where each method is noteworthy in producing narrower bands. We shall expand the restriction to (0.02, 0.98) in the data application in Sect. 6 to demonstrate the adequacy of the selected methods when approaching the boundaries of the entire time interval. Finally, nominal confidence level is set at 95% for all scenarios.

Each replicate simulation produces estimated bands $\{\widehat{\mathfrak {L}}_r(t), \widehat{\mathfrak {U}}_r(t)\}$ for $r = 1, \ldots , R$. We then compute the empirical coverage (EC) and average area between bands (AABB) as

$$\begin{aligned} EC&= R^{-1}\sum _{r=1}^{R}\mathbb {I}(\widehat{\mathfrak {L}}_r(t) \le S(t) \le \widehat{\mathfrak {U}}_r(t) \quad \forall t \in \mathcal {T}_{a,b}) \text { and}\\ AABB&= R^{-1}\sum _{r=1}^{R} \int _{\mathcal {T}_{a,b}} \left\{ \widehat{\mathfrak {U}}_r(t) -\widehat{\mathfrak {L}}_r(t)\right\} dt \text {, respectively,} \end{aligned}$$

where $S(t) = e^{-t}$ is the survival function for $T_i$. Note that *S*(*t*) is decreasing and each $\{\widehat{\mathfrak {L}}_r(t), \widehat{\mathfrak {U}}_r(t)\}$ is a step function. Therefore, for each constant step $[t_i, t_{i+1}]$, EC is practically computed by checking $S(t_i) \le \widehat{\mathfrak {U}}_r(t_i)$ and $\widehat{\mathfrak {L}}_r(t_{i+1}-) \le S(t_{i+1})$, where $f(t-) = \lim _{x \nearrow t} f(x)$. These metrics for each of the five methods are reported in Table 1. Because HW tends to produce the largest AABB among the five methods, all areas are normalized by the HW areas under the same scenario (e.g., EP AABB divided by HW AABB).

As exhibited in Table 1, and also originally noted by Nair (1984) and Hollander et al. (1997), the HW bands tend to be more conservative and have a larger area between bands than the other four methods do. For 0% censoring, OptBand, FD-I, EL, and EP attain nominal confidence level and are highly competitive with each other in terms of area between bands, with OptBand outperforming in terms of AABB in most restriction and sample size combinations under no censoring. In general, we see that EP bands are less competitive when the restriction is of the form $\mathcal {T}_{0.05, 0.80}$ or $\mathcal {T}_{0.20, 0.80}$. This is a well-known phenomenon, as EP bands are noted to have narrower tails than HW bands, but the advantage is diminished as bands are constructed on intervals bounded away from the tails. At 20% censoring, OptBand outperforms EL bands in most scenarios and at 50% censoring, OptBand generally outperforms whenever the restriction does not include the tails (e.g., $\mathcal {T}_{0.05, 0.80}$ or $\mathcal {T}_{0.20, 0.80}$) or under larger sample sizes. In general, EL bands outperform OptBand for smaller sample sizes, higher censoring, and restrictions including more of the tail ends of the survival curves. For the 20% and 50% censoring scenarios, OptBand and FD-I bands consistently produce at or nearly at nominal confidence level for all settings, with FD-I bands generally having less area between bands in smaller sample sizes and OptBand having less area in larger sample sizes. At 90% censoring, FD-I bands are conservative, while OptBand maintains nominal confidence level and produces less area between bands than FD-I does except in small sample sizes $(n = 100)$ and with the restrictions of the form $\mathcal {T}_{0.05, 0.80}, \mathcal {T}_{0.05, 0.95}$. Based on these observations, when censoring is very high and sample size is small, we recommend the use of FD-I. For larger sample sizes, we recommend the use of OptBand.

**Table 1:** Simulation results among the five methods comparing empirical coverage levels and average area between bands

			(Empirical coverage, average area between bands)				
*p*	*r*	*n*	OptBand	FD-I	EL	EP	HW
0	(.05, .95)	100	(.940, .854)	(.953, .886)	(.970, .882)	(.948, .895)	(.974, 1)
		500	(.942, .850)	(.949, .969)	(.967, .884)	(.955, .885)	(.959, 1)
		1000	(.942, .849)	(.940, .979)	(.964, .882)	(.952, .884)	(.952, 1)
	(.05, .8)	100	(.952, .961)	(.946, .927)	(.963, .989)	(.960, 1.01)	(.961, 1)
		500	(.952, .964)	(.949, .971)	(.947, 1.01)	(.958, 1.01)	(.958, 1)
		1000	(.949, .964)	(.949, .981)	(.948, 1.01)	(.949, 1.01)	(.958, 1)
	(.2, .95)	100	(.943, .853)	(.953, .889)	(.971, .863)	(.934, .876)	(.970, 1)
		500	(.950, .847)	(.952, .969)	(.965, .865)	(.963, .866)	(.961, 1)
		1000	(.952, .844)	(.957, .979)	(.967, .866)	(.961, .864)	(.966, 1)
	(.2, .8)	100	(.961, .973)	(.942, .935)	(.955, .976)	(.963, .996)	(.962, 1)
		500	(.940, .973)	(.945, .973)	(.955, .990)	(.949, .995)	(.953, 1)
		1000	(.948, .972)	(.946, .981)	(.958, .991)	(.951, .994)	(.952, 1)
.2	(.05, .95)	100	(.942, .856)	(.959, .824)	(.973, .864)	(.950, .888)	(.972, 1)
		500	(.945, .839)	(.947, .869)	(.959, .866)	(.944, .869)	(.955, 1)
		1000	(.950, .838)	(.954, .877)	(.956, .864)	(.964, .867)	(.962, 1)
	(.05, .8)	100	(.953, .964)	(.938, .914)	(.961, .986)	(.954, 1.01)	(.965, 1)
		500	(.962, .965)	(.958, .967)	(.964, 1.00)	(.962, 1.01)	(.966, 1)
		1000	(.952, .966)	(.952, .973)	(.965, 1.01)	(.960, 1.01)	(.960, 1)
	(.2, .95)	100	(.942, .855)	(.936, .819)	(.963, .850)	(.949, .868)	(.955, 1)
		500	(.944, .834)	(.955, .859)	(.947, .845)	(.957, .848)	(.965, 1)
		1000	(.950, .832)	(.941, .866)	(.951, .849)	(.950, .846)	(.950, 1)
	(.2, .8)	100	(.962, .978)	(.944, .918)	(,961, .973)	(.967, .996)	(.972, 1)
		500	(.944, .975)	(.938, .957)	(.959, .988)	(.942, .994)	(.945, 1)
		1000	(.951, .975)	(.954, .964)	(.954, .989)	(.957, .993)	(.956, 1)
.5	(.05, .95)	100	(.953, .896)	(.959, .800)	(.974, .858)	(.950, .902)	(.961, 1)
		500	(.949, .830)	(.950, .835)	(.954, .842)	(.950, .850)	(.958, 1)
		1000	(.956, .819)	(.951, .822)	(.959, .837)	(.951, .840)	(.947, 1)
	(.05, .8)	100	(.958, .976)	(.961, .932)	(.967, .979)	(.948, 1.01)	(.962, 1)
		500	(.951, .970)	(.944, .995)	(.956, .998)	(.958, 1.00)	(.956, 1)
		1000	(.960, .970)	(.953, 1.01)	(.958, 1.00)	(.949, 1.00)	(.959, 1)
	(.2, .95)	100	(.957, .896)	(.963, .784)	(.963, .847)	(.954, .881)	(.957, 1)
		500	(.954, .823)	(.949, .787)	(.956, .820)	(.964, .826)	(.968, 1)
		1000	(.954, .814)	(.946, .794)	(.953, .815)	(.952, .819)	(.952, 1)
	(.2, .8)	100	(.959, .995)	(.950, .927)	(.959, .972)	(.964, 1.00)	(.966, 1)
		500	(.957, .982)	(.949, .969)	(.947, .986)	(.954, .992)	(.952, 1)
		1000	(.957, .981)	(.954, .981)	(.951, .988)	(.958, .991)	(.961, 1)
.9	(.05, .95)	100	(.908, .911)	(.983, 1.15)	(.955, .928)	(.860, .928)	(.940, 1)
		500	(.938, 1.03)	(.974, 1.49)	(.955, .967)	(.902, .955)	(.889, 1)
		1000	(.936, .988)	(.984, 1.57)	(.941, .939)	(.903, .923)	(.888, 1)
	(.05, .8)	100	(.908, .908)	(.988, 1.17)	(.952, .949)	(.859, .924)	(.948, 1)
		500	(.935, 1.04)	(.983, 1.39)	(.942, 1.01)	(.887, .995)	(.891, 1)
		1000	(.934, 1.01)	(.980, 1.36)	(.959, 1.01)	(.906, .994)	(.908, 1)
	(.2, .95)	100	(.954, .998)	(.982, 1.17)	(.957, .946)	(.925, .972)	(.960, 1)
		500	(.946, 1.04)	(.979, 1.42)	(.954, .967)	(.912, .945)	(.900, 1)
		1000	(.944, .991)	(.988, 1.46)	(.945, .928)	(.906, .909)	(.894, 1)
	(.2, .8)	100	(.944, 1.01)	(.993, 1.21)	(.955, .981)	(.919, .978)	(.950, 1)
		500	(.953, 1.08)	(.995, 1.34)	(.946, 1.04)	(.926, 1.02)	(.926, 1)
		1000	(.943, 1.04)	(.983, 1.25)	(.951, 1.02)	(.915, 1.01)	(.906, 1)

The average area between bands are all normalized by the HW areas. Here, $p =$ censoring proportion, $r =$ restriction interval $\mathcal {T}_r$, $n=$ sample size

## Primary biliary cirrhosis data analysis

The Mayo Clinic trial in primary biliary cirrhosis (PBC) of the liver, described in Appendix D of Fleming and Harrington (1991) and accessible in the *survival* package in R (Therneau 2015), was a double-blind, randomized, placebo-controlled study conducted between 1974 and 1984 to evaluate the efficacy of the treatment drug D-penicillamine. The primary endpoint was death, but some patients received a liver transplant. A total of 312 patients were enrolled in the randomized trial, with 158 given placebo and 154 given treatment. This dataset had an additional 112 non-randomized patients who consented to have basic measurements recorded and to be followed for survival; we excluded these patients in our application. The mean follow-up time for the randomized subjects was 5.5 years with approximately 50% censoring across groups. The original analysis found no benefit of D-penicillamine over placebo. For illustrative purposes, we fit survival curves and confidence bands for the D-penicillamine group, the placebo group, and over both groups pooled together and consider a liver transplant to be a censoring event.

**Fig. 1:**
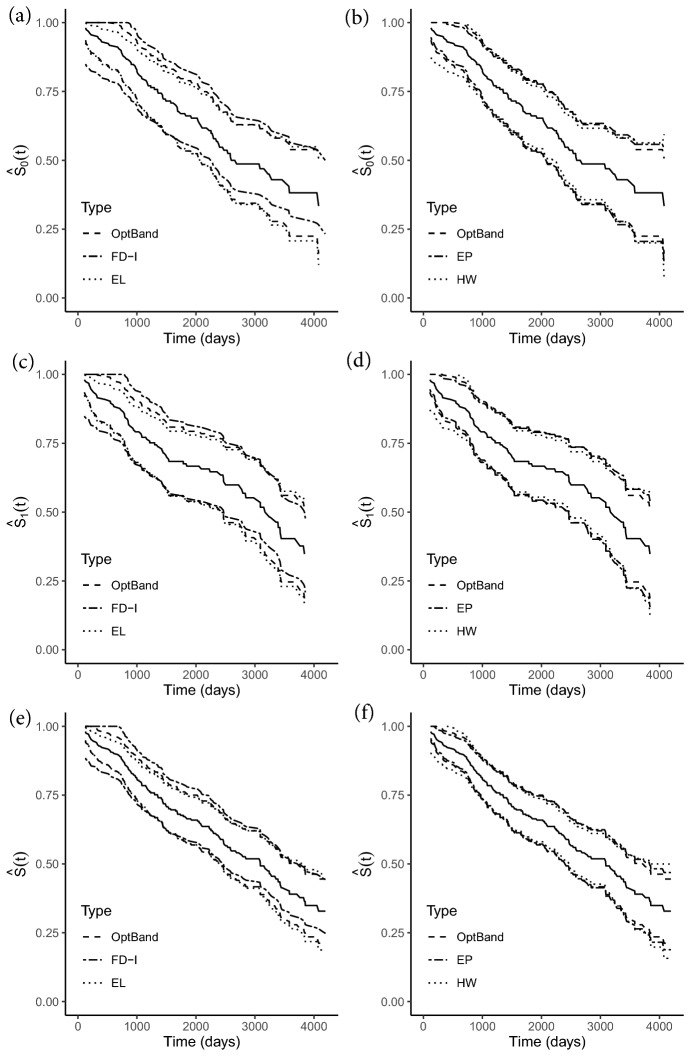
Survival plots for placebo group (**a**, **b**), D-penicillamine group (**c**, **d**), and pooling both groups together (**e**, **f**). 95% OptBand, Fiducial (FD-I), Empirical Likelihood (EL), Equal Precision (EP), and Hall-Wellner bands plotted over all combinations. OptBand is present on all plots for easier comparison

We construct OptBand, FD-I, EL, EP, and HW bands for time to endpoint on the D-penicillamine, placebo, and aggregated groups on the restricted interval $\mathcal {T}_{0.02, 0.98}$; as stated under Sect. 5, this is the largest interval for which critical values for EL and EP are available. Figure 1a–f compare OptBand against each of FD-I, EL, EP, and HW bands for the survival function in the placebo group, D-penicillamine group, and the two groups pooled together, respectively. Table 2 displays the normalized area between bands for the aforementioned scenarios. We observe that EL provides the least area between bands for placebo and D-penicillamine groups separately, while OptBand provides the least area for the pooled group. This is completely in line with our simulation conclusions, as EL outperforms when censoring is high ($\sim$50% or higher) and sample size is small ($\sim$100), which match the characteristics of the placebo or D-penicillamine groups separately. Combining the two groups, the sample size becomes sufficiently large such that optimality properties of OptBand become more pronounced.

**Fig. 2:**
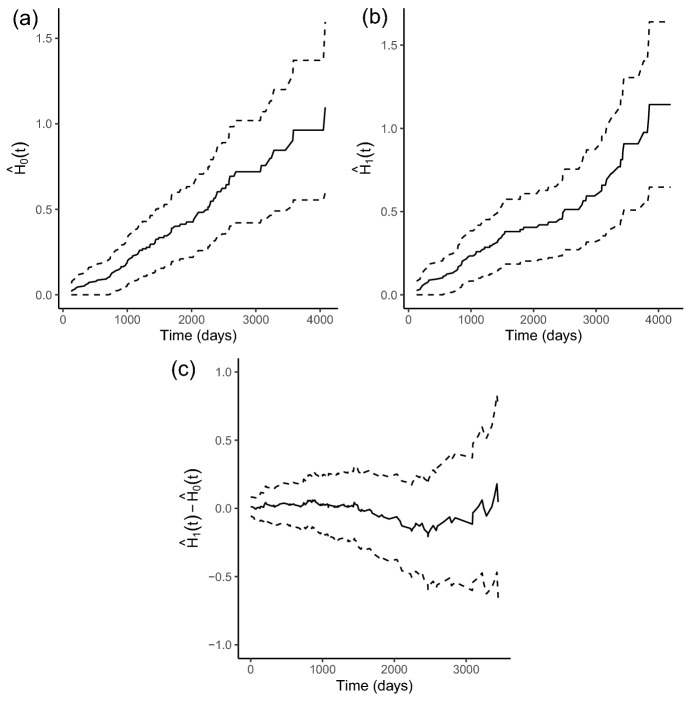
OptBand applied to the placebo (**a**) and then the D-penicillamine (**b**) group of the PBC trial data to compute 95% CBs for the cumulative hazard functions, as well as for the difference in cumulative hazard functions between the two groups (**c**)

We construct OptBand for the difference in cumulative hazard functions, as seen in Fig. 2, where 2a and b depict the 95% CBs for the placebo and treatment groups, respectively, while 2c depicts the difference between cumulative hazard functions of the two groups. The bands suggest a lack of evidence for a significant difference in cumulative hazard functions between the two groups at the 0.05 significance level.

## Discussion

In this work, we present analytical CBs for the one-sample cumulative hazard function, two-sample difference in cumulative hazard functions, and one-sample survival functions which approximately minimize the area between bands and hence approximates the HCDR. Classical bands such as HW and EP require only a table of critical values and hence are fast to compute, albeit the larger area between bands. EL and fiducial methods, while possibly providing lower area, can be computationally intensive. OptBand strikes a delicate balance between computational simplicity and roughly targeting the HCDR. Intuitively, one would expect a CB to take the form of an estimator $\widehat{\theta }(t)$ plus or minus some variation $\alpha (t)\cdot \text {se}(\widehat{\theta }(t))$. OptBand takes exactly this form, with $\alpha (\cdot )$ encompassing the $\psi (\cdot )$ function within. Hence, the $\psi (\cdot )$ function can be viewed as the appropriate weighting function that shapes the bands to have approximately minimal area.

**Table 2:** Area between bands, normalized by Hall-Wellner area, for each band type and each grouping of the PBC data

			Average area between bands				
Group	*p*	*n*	OptBand	FD-I	EL	EP	HW
Placebo	.535	158	.980	1.035	.964	.981	1
D-penicillamine	.563	154	.987	1.044	.971	.989	1
Overall	.558	312	.957	1.030	.960	.970	1

Here, *p* = censoring proportion, *n* = sample size

Unfortunately, designing OptBand for the risk difference $S_2(t) - S_1(t)$ or ratio $S_2(t)/S_1(t)$, at least with our current framework derived from Kendall et al. (2007), remains an intractable problem. The difference

$$\begin{aligned}{}[S_2(t) - S_1(t)] - [\widehat{S}_2(t) - \widehat{S}_1(t)] \end{aligned}$$

can be shown to converge weakly to $S_1(t)W(\sigma _1^2(t)) + S_2(t)W(\sigma ^2_2(t))$, which cannot be further manipulated into a form befitting derivation of OptBand; HW and EP bands face a similar intractability for the difference of survival curves. Bands for the ratio face a different intractability. Since $H_1(t) - H_2(t) = \log (S_2(t)/S_1(t))$, we have that

$$\begin{aligned} \frac{\widehat{S}_2(t)/\widehat{S}_1(t) - S_2(t)/S_1(t)}{S_2(t)/S_1(t)} \end{aligned}$$

weakly converges to $W(\sigma _1^2(t) + \sigma _2^2(t))$, and hence at first glance OptBand for the ratio could be derived in a similar manner to Optband for *S*(*t*) in the one-sample problem. However, in the derivation for *S*(*t*), we rely on the non-increasing monotonicity of *S*(*t*) (see Appendix B for how this fact is utilized), but $S_2(t)/S_1(t)$ is no longer monotone. We hope to derive bands for the ratio, and in general, functions of $S_1(t)$ and $S_2(t)$, in future works.

Further work expanding OptBand to conditionally independent censoring scenarios would be interesting. Given treatment indicator $A \in \{0, 1\}$ and baseline covariates such that *T* and *C* are independent given *A* and *X*, one could fit a Cox model

$$\begin{aligned} h(t|A, X) = h_A(t) e^{\beta ^\intercal X} \end{aligned}$$

where the proportional hazards assumption is imposed on *X*, but the baseline hazards do not need to be proportional between treatments. In testing the null hypothesis $H_0(t) = H_1(t)$ for all $t \in [t_L, t_U]$ vs the alternative $H_0(t) \ne H_1(t)$ for some $t \in [t_L, t_U]$, OptBand can be extended to the Breslow estimators for $H_0(t)$ and $H_1(t)$. Using the fact that the Breslow estimators are asymptotically Brownian, we may compute confidence intervals for $H_1(t) - H_0(t)$ in the exact same manner as seen in Sect. 3.2.

These methods have been published for use in the R package *optband* on CRAN. We recommend the use of OptBand with some minor restrictions of the event times (e.g., $\mathcal {T}_{0.05, 0.95}$). Our experiences with OptBand show that it is quite robust to censoring, even at 90% censoring.

## Figures and Tables

**Fig. 3:**
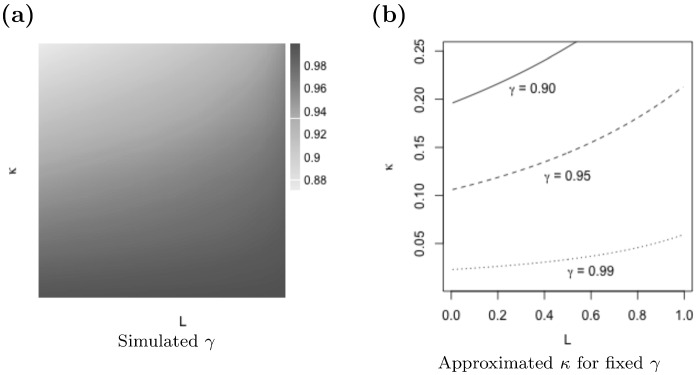
Simulated coverage in terms of $\kappa$ (vertical axis) and *L* (horizontal axis). This smooth relationship (**a**) is closely matched by a functional approximation (**b**)

## A Minimal-area bands from Kendall et al. (2007)

Below are the results specified by Kendall et al. (2007) with a bit more generality. Suppose we intend to find a continuous function *u* on $[t_L, t_U]$ that is a solution to the following optimization problem:

$$\begin{aligned} \min _{u}\left( \int _{t_L}^{t_U}u(t) dt\right) \quad \text {s.t.} \quad \mathbb {P}(-u(t) \le W(\sigma ^2(t)) \le u(t) \quad \forall t \in [t_L, t_U]) = \gamma , \end{aligned}$$

where $W(\sigma ^2(t))$ is a zero-mean Weiner process with strictly increasing variance function $\sigma ^2(t)$. We call $\gamma$ the *coverage level*, and it is more commonly thought of as $\gamma = 1 - \alpha$, where $\alpha$ is the *significance level*. The objective to be minimized is clearly one-half the area between the bands, which is exactly the objective of finding the HCDR. We may consider the dual problem

$$\begin{aligned} \min _{u} 1 - \mathbb {P}(-u(t) \le W(\sigma ^2(t)) \le u(t) \quad \forall t \in [t_L, t_U]) \quad \text {s.t.} \; \left( \int _{t_L}^{t_U}u(t) dt\right) = \beta , \end{aligned}$$

where $\beta$ is a constant related to $\gamma$. Due to the intractability of the objective function, the key observation Kendall et al. noted was to consider a related problem based on local times:

$$\begin{aligned} \min _{u} \mathbb {E}[L^u[t_L, t_U] + L^{-u}[t_L, t_U]] \quad \text {s.t.} \quad \left( \int _{t_L}^{t_U}u(t) dt\right) = \beta , \end{aligned}$$

where $L^u[t_L, t_U]$ is the local time accumulated by $W(\sigma ^2(t))$ along curve *u* on the interval $[t_L, t_U]$. We can calculate

$$\begin{aligned} \mathbb {E}L^u[t_L, t_U]&= \mathbb {E}\lim _{\epsilon \rightarrow 0} \frac{1}{\epsilon }\int _{t_L}^{t_U} \mathbb {I}\{u(t) \le W(\sigma ^2(t)) < u(t) + \epsilon \} dt \\&= \frac{1}{\sqrt{2\pi }}\int _{t_L}^{t_U} \exp \left( -\frac{u(t)^2}{2\sigma ^2(t)}\right) \frac{1}{\sigma (t)} dt \end{aligned}$$

and similarly for $\mathbb {E}L^{-u}[t_L, t_U]$. So we ultimately have

$$\begin{aligned} \min _{u} \frac{2}{\sqrt{2\pi }}\int _{t_L}^{t_U} \exp \left( -\frac{u(t)^2}{2\sigma ^2(t)}\right) \frac{1}{\sqrt{\sigma ^2(t)}} dt \quad \text {s.t.} \quad \left( \int _{t_L}^{t_U}u(t) dt\right) = \beta , \end{aligned}$$

which can be solved through the Euler-Lagrange equation to obtain

$$\begin{aligned} u_\kappa ^*(t) = \psi (\kappa \sigma ^2(t))\sigma (t). \end{aligned}$$

For all combinations of $(\kappa , L)$ from $\kappa = 0.01, 0.02, \ldots , 0.25$ and $L= 0.005,0.010, \ldots ,0.995$, we estimated $\gamma =1-\alpha$ with

$$\begin{aligned} \widehat{\gamma }_{\kappa , L} = N^{-1}\sum _{n=1}^{N}\mathbb {I}\Bigg (&|\widehat{W}_{\tau , n}(s)| \le \psi (\kappa s)\sqrt{s} \quad \forall s = \left\{ L, L+\frac{1}{\tau }, \ldots , 1 - \frac{1}{\tau }, 1\right\} \Bigg ) \end{aligned}$$

where $\widehat{W}_{\tau , n}(t) = \frac{1}{\sqrt{\tau }}\sum _{k=1}^{\tau }\sqrt{t} Z_{k,n}$, with $Z_{k,n}$ being i.i.d. standard normal, and $t = \frac{1}{\tau }, \cdots , 1$. This provides an approximation to the standard Brownian motion on the unit interval, with the step-size parameter $\tau$ controlling how granular the approximated process is and *N* being the number of Monte Carlo simulations. In our simulations, $N = 4\times 10^5$ and $\tau = 10^5$. Note that this choice of $\tau$ partitions the interval [*L*, 1] into intervals of equal size, given the sequence for *L*. We further justify our functional approximation with a heatmap in Fig. 3. The borders between the colors on the heatmap are smooth and closely match up with the $\gamma$ contours defined from our functional approximation $\widehat{\gamma }_{\kappa , L} = 1 + (a+bL)\kappa + a\kappa ^2$ in Eq. (4) proposed in the main text. We chose this specific functional approximation based on empirical observations. We first noticed by setting $L = 0$, the coefficients of $\kappa$ and $\kappa ^2$ were nearly identical in the quadratic fit of $\kappa$ and $\hat{\gamma }_{\kappa , 0}$. Next, we noticed that the coefficient of $\kappa$ increased linearly and the coefficient of $\kappa ^2$ remained nearly constant with respect to *L*, which is exactly described in the functional approximation above. Of course, one could include more parameters for marginal increases in accuracy, but in favor of parsimony, we selected this relatively simple two-parameter approximation.

## B derivation of OptBand for the survival function

In order to exactly target the bands around $\widehat{S}(t) - S(t)$, we may consider the optimization problem

$$\begin{aligned} \min _{u} \frac{2}{\sqrt{2\pi }}\int _{t_L}^{t_U} \exp \left( -\frac{u(t)^2}{2\sigma ^2(t)}\right) \frac{1}{\sigma (t)} dt \quad \text {s.t.} \quad \left( \int _{t_L}^{t_U}S(t)u(t) dt\right) = \beta \end{aligned}$$

which, through similar steps taken in Appendix A, gives us the solution

$$\begin{aligned} u^*(t) = \psi (\kappa S(t)\sigma ^2(t))\sigma (t). \end{aligned}$$

Again, making the transformation $\sigma ^2(t) \mapsto \sigma ^2(t)/\sigma ^2(t_U)$, we find $\kappa$ so that the event

$$\begin{aligned} |W\left( s\right) | \le \psi \left( \kappa S(t)s\right) \sqrt{s} \quad \forall s \in \left[ \frac{\sigma ^2(t_L)}{\sigma ^2(t_U)}, 1\right] \end{aligned}$$

occurs with probability $\gamma$, where $t = [\sigma ^2]^{-1}\left\{ \sigma ^2(t_U)s\right\}$ and $[\sigma ^2]^{-1}$ denotes the inverse variance function. We now face a problem where our $\kappa$ is also a function of *S*(*t*). We propose the computation scheme. Denote

$$\begin{aligned} \mathcal {E}(\eta (\cdot ), \tau , \upsilon ) = \left\{ \left| W\left( \frac{\sigma ^2(t)}{\sigma ^2(t_U)}\right) \right| \le \psi \left( \eta (t)\frac{\sigma ^2(t)}{\sigma ^2(t_U)}\right) \frac{\sigma (t)}{\sigma (t_U)} \quad \forall t \in [\tau , \upsilon ]\right\} . \end{aligned}$$

Now if $\eta (\cdot ) = \eta$ is a constant, we can approximate this quantity with Eq. (4):

$$\begin{aligned} \mathbb {P}(\mathcal {E}(\eta , \tau , \upsilon ))&= \mathbb {P}\left( \left| W\left( \frac{\sigma ^2(t)}{\sigma ^2(\upsilon )}\right) \right| \le \psi \left( \eta \frac{\sigma ^2(\upsilon )}{\sigma ^2(t_U)}\frac{\sigma ^2(t)}{\sigma ^2(\upsilon )}\right) \frac{\sigma (t)}{\sigma (\upsilon )} \quad \forall t \in [\tau , \upsilon ]\right) \\&\approx 1 + \left( a+b\frac{\sigma ^2(\tau )}{\sigma ^2(\upsilon )}\right) \left( \eta \frac{\sigma ^2(\upsilon )}{\sigma ^2(t_U)}\right) + a\left( \eta \frac{\sigma ^2(\upsilon )}{\sigma ^2(t_U)}\right) ^2. \end{aligned}$$

In what follows, we will need the fact

6 $$\begin{aligned} \begin{aligned} \eta _1 \le \eta _2&\implies \mathcal {E}(\eta _1, \tau , \upsilon ) \supseteq \mathcal {E}(\eta _2, \tau , \upsilon ) \end{aligned} \end{aligned}$$

which follows, since a larger $\eta$ corresponds to tighter bands.

Let $t_L = \xi _1 \le \xi _2 \le \cdots \le \xi _K = t_U$ be some partition of the interval $[t_L, t_U]$, with the most natural choice being the observed event time points $\{t_i: t_L \le t_i \le t_U\}$. Let $\overline{S}_i = (\widehat{S}(\xi _i) + \widehat{S}(\xi _{i+1}))/2$. The construction of OptBand for survival functions is based on two approximations: (1) approximate *S*(*t*) for $t \in [\xi _i, \xi _{i+1}]$ with a midpoint Riemann sum $\overline{S}_i$, (2) approximate with Eq. (4) for each locally constant portion of $\overline{S}_i$. More specifically, the first approximation is

7 $$\begin{aligned} \begin{aligned} \gamma = \mathbb {P}\left( \bigcap _{i=1}^{K-1}\mathcal {E}(\kappa S(\cdot ), \xi _i, \xi _{i+1})\right) \approx \mathbb {P}\left( \bigcap _{i=1}^{K-1}\mathcal {E}(\kappa \overline{S}_i, \xi _i, \xi _{i+1})\right) \end{aligned} \end{aligned}$$

with the approximation increasingly accurate as $K \rightarrow \infty$. In practice, there are no advantages in choosing $\{\xi _1, \cdots , \xi _K\}$ to be anything else but the observed event times because only at these values does the estimated survival function $\widehat{S}(\cdot )$ change, and therefore the Riemann sum cannot become more accurate than that.

The interpretation of $\bigcap _{i=1}^{K-1}\mathcal {E}(\kappa \overline{S}_i, \xi _i, \xi _{i+1})$ is the event that our Brownian motion falls within our bands on interval $[\xi _i, \xi _{i+1}]$ for each *i*. Equivalently, since $\kappa \overline{S}_i$ is non-increasing, hence $\mathcal {E}(\kappa \overline{S}_i, \xi _{i+1}, \xi _{K})$ are tighter than $\mathcal {E}(\kappa \overline{S}_{i+1}, \xi _{i+1}, \xi _{K})$ by Eq. (6), we can imagine first having to fall within $\mathcal {E}(\kappa \overline{S}_1, \xi _{1}, \xi _{K})$, then next having to fall within $\mathcal {E}(\kappa \overline{S}_2, \xi _{2}, \xi _{K})$, etc, leading to the equivalence

$$\begin{aligned} \bigcap _{i=1}^{K-1}\mathcal {E}(\kappa \overline{S}_i, \xi _i, \xi _{i+1}) = \bigcup _{i=1}^{K-1}\mathcal {E}(\kappa \overline{S}_i, \xi _i, \xi _{K}). \end{aligned}$$

Continuing Eq. (7) and defining $\mathcal {E}(\kappa \overline{S}_{0}, \xi _{0}, \xi _{K}) = \emptyset$, we have

$$\begin{aligned} \gamma&\approx \mathbb {P}\left( \bigcup _{i=1}^{K-1}\mathcal {E}(\kappa \overline{S}_i, \xi _i, \xi _{K})\right) \\&= \mathbb {P}\left( \bigcup _{i=1}^{K-1}\left[ \mathcal {E}(\kappa \overline{S}_i, \xi _i, \xi _{K})\setminus \mathcal {E}(\kappa \overline{S}_{i-1}, \xi _{i}, \xi _{K})\right] \right) \\&= \sum _{i=1}^{K-1}\left\{ \mathbb {P}\left( \mathcal {E}(\kappa \overline{S}_i, \xi _i, \xi _{K})) - \mathbb {P}(\mathcal {E}(\kappa \overline{S}_{i-1}, \xi _{i}, \xi _{K})\right) \right\} \\&\approx \frac{\kappa b}{\sigma ^2(t_U)}\left\{ \sum _{i=1}^{K-2}\overline{S}_i(\sigma ^2(\xi _i) - \sigma ^2(\xi _{i+1}))\right\} + 1 + \left( a + b \frac{\sigma ^2(\xi _{K-1})}{\sigma ^2(t_U)}\right) (\kappa \overline{S}_{K-1}) \\&\quad + a(\kappa \overline{S}_{K-1})^2 \end{aligned}$$

where the last $\approx$ comes from continued application of Eq. (4). This is a quadratic function in $\kappa$, and hence we can solve for $\kappa$ analytically. We also estimate $\sigma ^2(t)$ with $\widehat{\sigma }^2(t)$, as before. Finally, we pivot to get a $100\gamma \%$ CI:

$$\begin{aligned} \gamma&\approx \mathbb {P}\left( \left| n^{1/2}\frac{\widehat{S}(t) - S(t)}{\widehat{S}(t)\widehat{\sigma }(t_U)}\right| \le \psi \left( \kappa \widehat{S}(t)\frac{.\widehat{\sigma }^2(t)}{.\widehat{\sigma }^2(t_U)}\right) \frac{\widehat{\sigma }(t)}{\widehat{\sigma }(t_U)} \quad \forall t \in [t_L, t_U]\right) \\&= \mathbb {P}\left( \widehat{S}(t)\left( 1 - c_S(t) \right) \le S(t) \le \widehat{S}(t)\left( 1 + c_S(t) \right) \quad \forall t \in [t_L, t_U]\right) , \end{aligned}$$

where $$c_S(t) = \psi \left( \kappa \widehat{S}(t)\frac{\widehat{\sigma }^2(t)}{\widehat{\sigma }^2(t_U)}\right) \frac{\widehat{\sigma }^2(t)}{n^{1/2}}$$.
